# Mapping the Internal Anatomy of the Lateral Brainstem: Anatomical Study with Application to Far Lateral Approaches to Intrinsic Brainstem Tumors

**DOI:** 10.7759/cureus.1010

**Published:** 2017-02-03

**Authors:** Joe Iwanaga, Andre Granger, Payman Vahedi, Marios Loukas, Rod J Oskouian, Fabian N Fries, Iraj Lotfinia, Martin M Mortazavi, W. Jerry Oakes, R. Shane Tubbs

**Affiliations:** 1 Seattle Science Foundation; 2 Department of Anatomical Sciences, St. George's University School of Medicine, Grenada, West Indies; 3 Neurological Surgery, Thomas Jefferson University; 4 Neurosurgery, Complex Spine, Swedish Neuroscience Institute; 5 Saarland University Faculty of Medicine, Saarland University Medical Center; 6 Neurosurgery, Tabriz University of Medical Sciences; 7 Department of Neurosurgery, University of Washington School of Medicine, Seattle, WA; 8 Pediatric Neurosurgery, Children’s of Alabama; 9 Neurosurgery, Seattle Science Foundation

**Keywords:** anatomy, neurosurgery, approaches, nuclei, morbidity

## Abstract

**Introduction:**

Intramedullary brainstem tumors present a special challenge to the neurosurgeon. Unfortunately, there is no ideal part of the brainstem to incise for approaches to such pathology. Therefore, the present study was performed to identify what incisions on the lateral brainstem would result in the least amount of damage to eloquent tracts and nuclei. Case illustrations are also discussed.

**Materials and methods:**

Eight human brainstems were evaluated. Based on dissections and the use of standard atlases of brainstem anatomy, the most important deeper brainstem structures were mapped to the surface of the lateral brainstem.

**Results:**

With these data, we defined superior acute and inferior obtuse corridors for surgical entrance into the lateral brainstem that would minimize injury to deeper tracts and nuclei, the damage to which would result in significant morbidity.

**Conclusions:**

To our knowledge, a superficial map of the lateral brainstem for identifying deeper lying and clinically significant nuclei and tracts has not previously been available. Such data might decrease patient morbidity following biopsy or tumor removal or aspiration of brainstem hemorrhage. Additionally, this information can be coupled with the previous literature on approaches into the fourth ventricular floor for more complex, multidimensional lesions.

## Introduction

Historically, brainstem tumors have been notorious for the challenges that they bring to the neurosurgeon; however, with the advent of advanced neuroimaging and intraoperative electrophysiological neuromonitoring in recent years, there's a growing concept that carefully selected brainstem tumors could be safely approached. Nevertheless, the complexity in the neuroanatomy and functionality has impeded the development of microsurgical techniques in this area [1]. The close relationship that these tumors share with critical areas of the central nervous system largely contributes to the morbidity and mortality that are sometimes associated with them and their management [2-4]. Gaining access to and obtaining satisfactory resection of these abnormal growths provide a unique array of difficulties to the neurosurgeon. Approaches include retrosigmoid, suboccipital (with or without telovelar approach), supracerebellar infratentorial, orbitozygomatic, far lateral [5], subtemporal transtentorial (for anterolateral and upper pons tumors), pterional transsylvian (for median ventral surface tumors of midbrain) and combined petrosal (for focal ventrolateral pontine tumors). In this article, we look at the far lateral approach to brainstem tumors and the related brainstem anatomy. A cadaveric study was performed to best define anatomical relationships of the lateral brainstem in order to minimize surgical morbidity. In addition, a case illustration utilizing the lateral approach and the literature reviewed for other surgical approaches to the lateral brain stem are included. Informed consent was obtained from the patient for this study.

## Materials and methods

**Figure 1:**
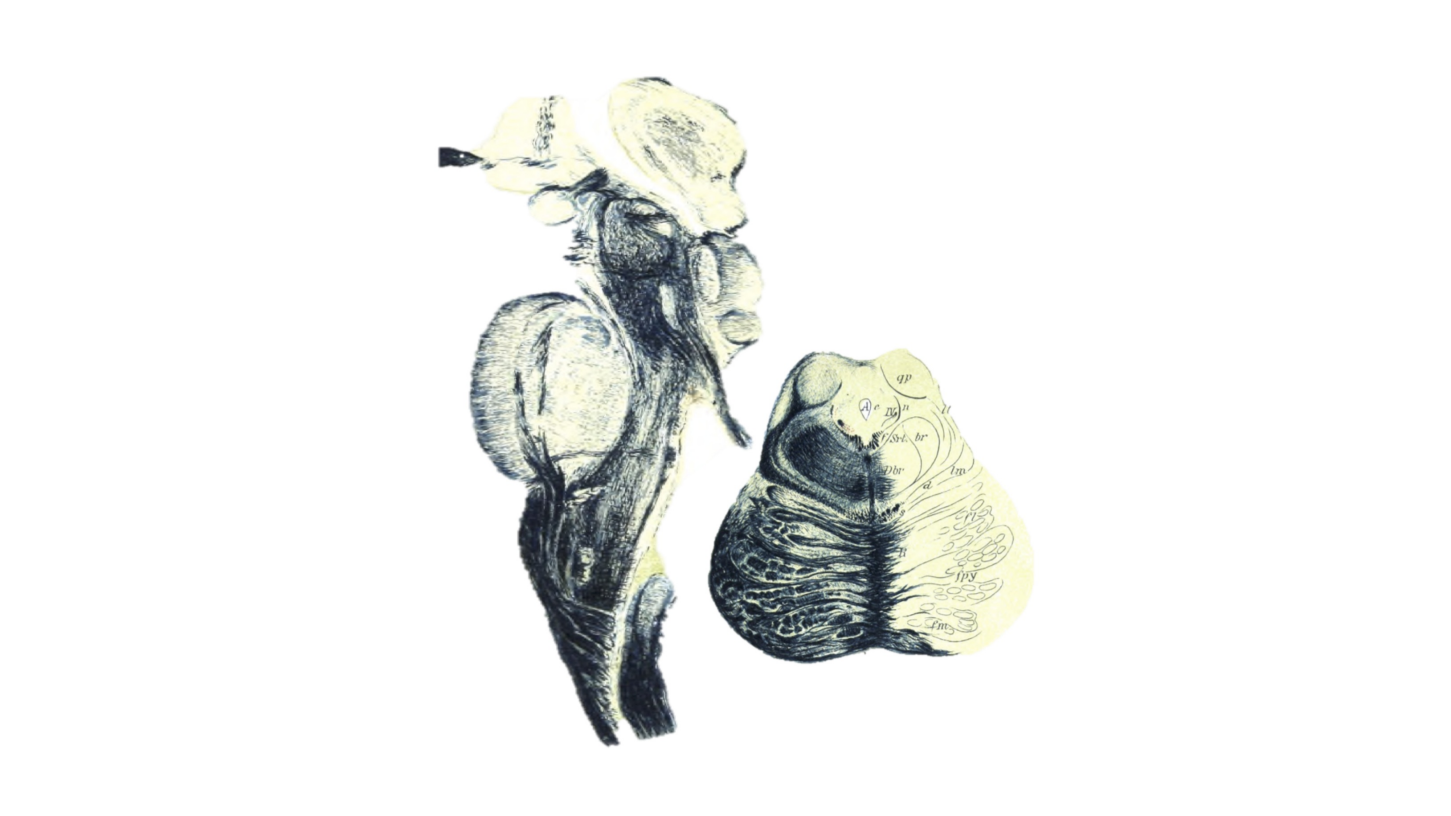
Sagittal section of the brainstem (left) with example axial section through the pons (right). Atlas of the Nervous System by Christfried Jakob (Philadelphia: WB Saunders, 1901).

**Figure 2:**
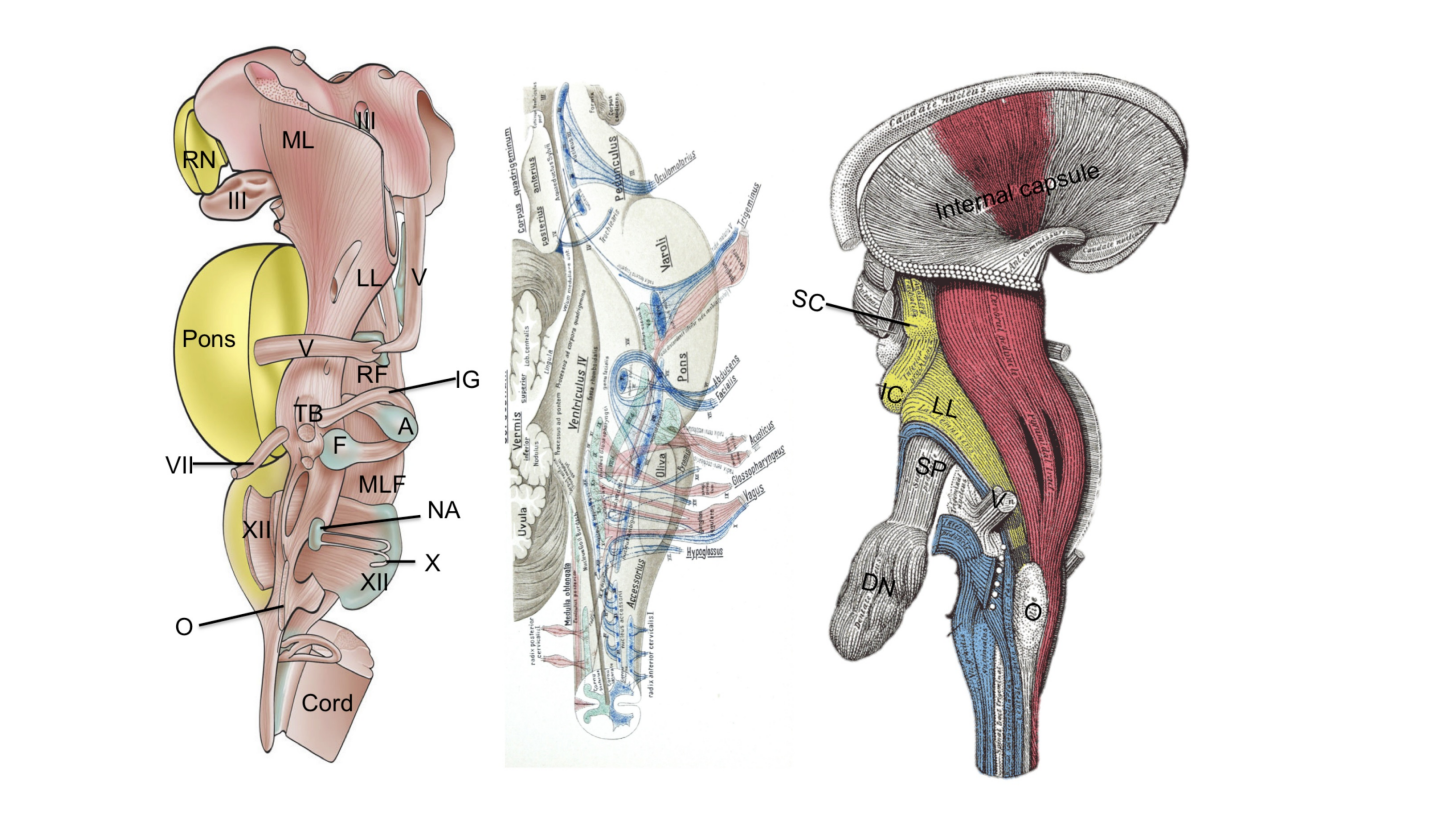
Three examples of deeper neural structures within the brainstem. The image to the left illustrates the red nucleus (RN), medial lemniscus (ML), oculomotor nucleus and nerve (III), lateral lemniscus (LL), tracts of the trigeminal nerve (V), reticular formation (FGF), trapezoid body (TB), abducens nucleus (A), facial nucleus (F), internal genu (IG), facial nerve (VII), medial longitudinal fasciculus (MLF), hypoglossal nucleus and fibers (XII), dorsal vagal nucleus (X), olive (O), and distally, the spinal cord (cord). The middle panel (Jacob’s Atlas) illustrates the various cranial nerve tracts of the brainstem and the figure to the right (Gray’s Anatomy) illustrates, primarily, the corticospinal tract and its relationship within the brainstem. Also noted in this figure are the superior colliculus (SC), inferior colliculus (IC), lateral lemniscus (LL), superior cerebellar peduncle (SP), dentate nucleus (DN) trigeminal nerve (V) and olive (O). The solitary nucleus is approximated by the dotted line shown for the dorsal vagal nucleus.

**Figure 3:**
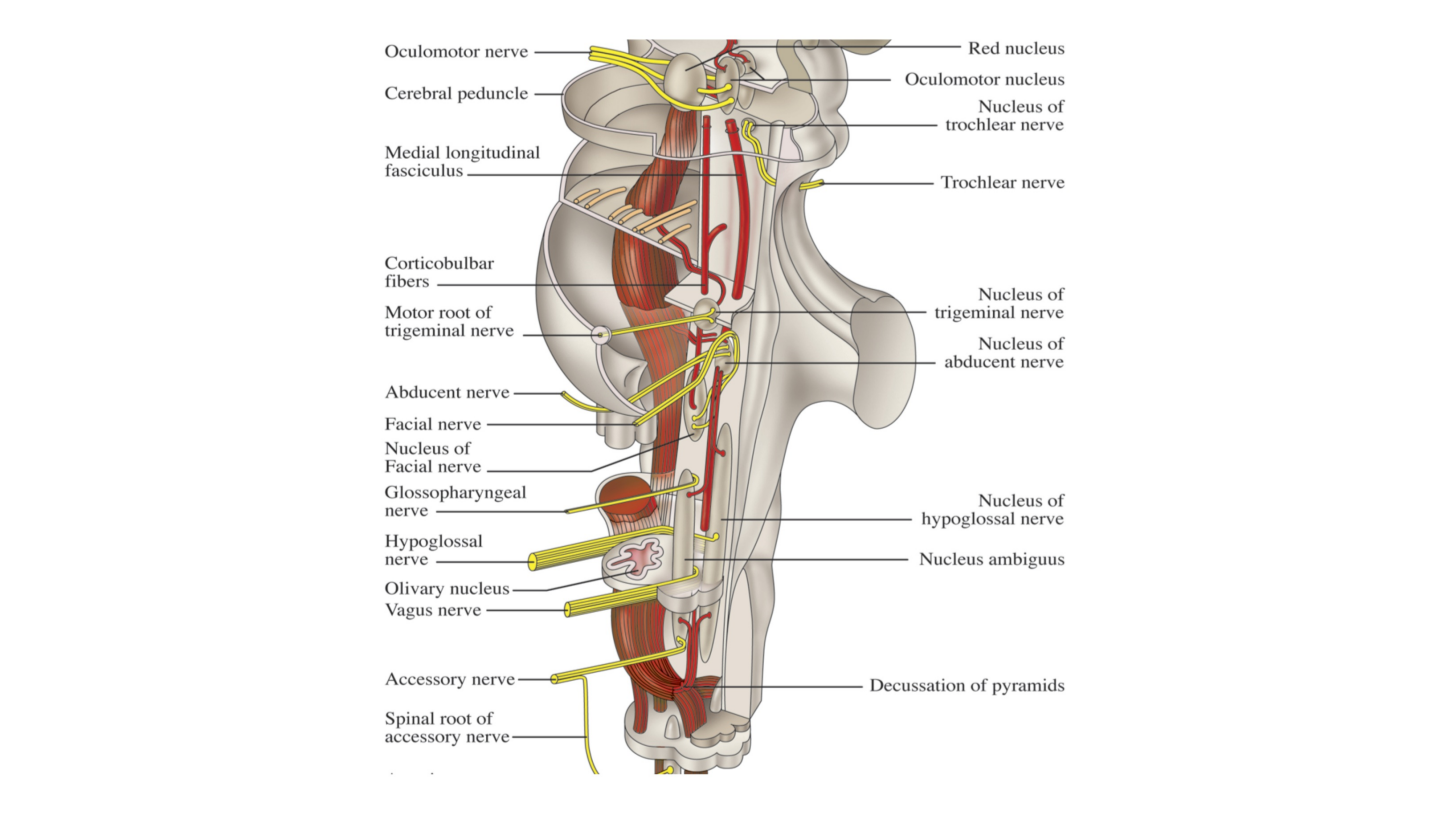
Schematic drawing of the lateral brainstem illustrating some of the major nuclei and tracts.

Eight human formalin-fixed brainstems were evaluated. These were derived from four male and four female specimens with an age at death of 41 to 87 years (mean 78 years). With cadavers in the supine position, the calvaria were removed with an oscillating bone saw (Stryker, MI, USA). The dura mater was opened using dissecting scissors and the cerebrum removed with a scalpel. The spinal cord was then transectioned by cutting it with a #15 scalpel blade attached to a long scalpel handle that was introduced anterior to the brainstem and into the upper spinal canal. In addition, the first two denticulate ligaments on the left and right sides were cut. The intact brainstem beginning at the midbrain and ending with the medulla oblongata was then removed and sectioned every 3 mm (four specimens were sectioned axially, two specimens were sectioned coronally, and two specimens were sectioned sagittally) into 5-micron segments on a microtome. Sliced segments were applied to slides and stained (hematoxylin and eosin (H&E), Weigert). Under the microscope (4-10x magnification) and using reference localization per Jakob’s *Atlas of the Nervous System*, critical nuclei and tracts were identified and mapped to the lateral surface of the brainstem (Figures 1-3).

**Figure 4:**
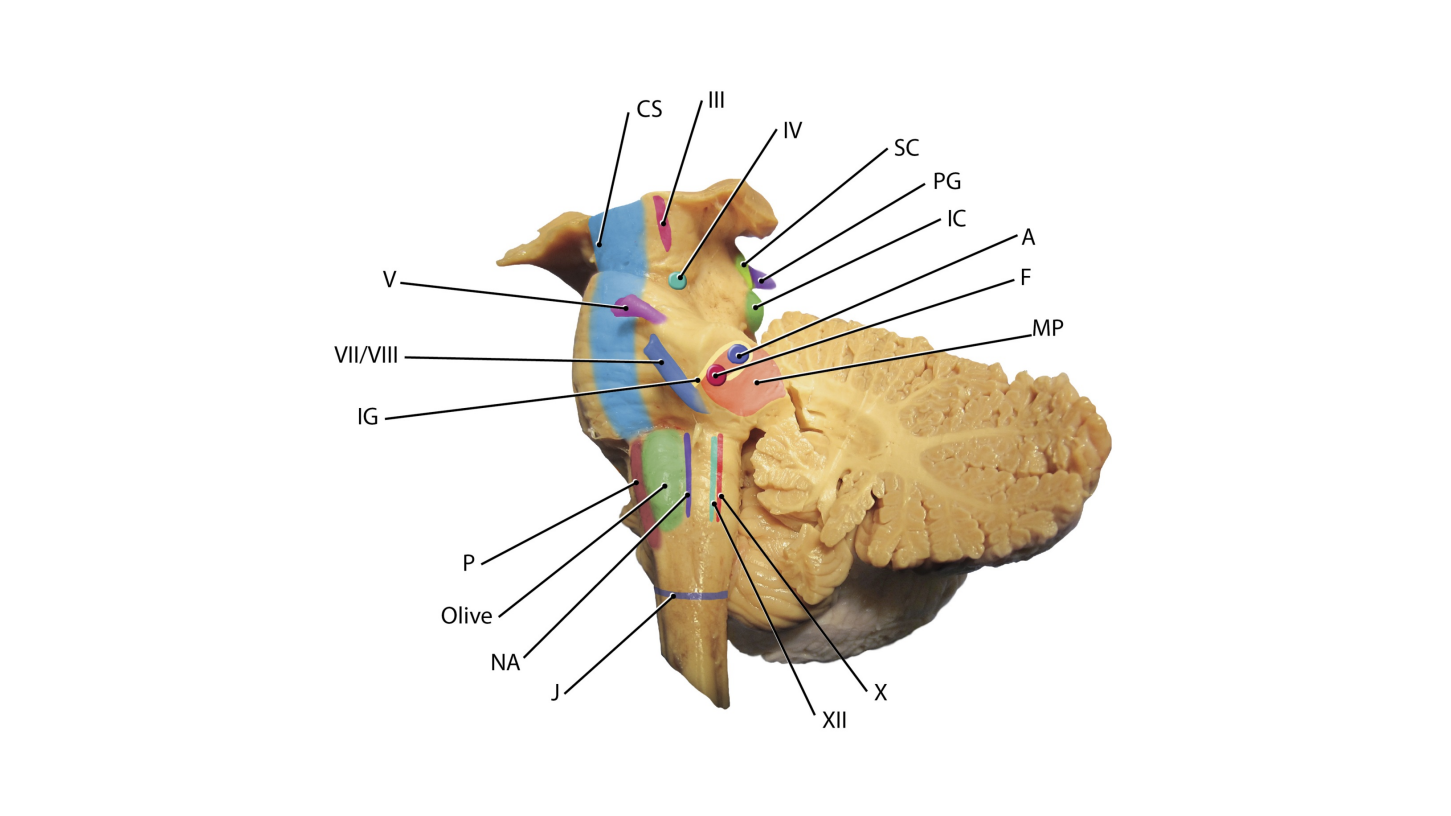
Posterolateral view of the brainstem with left cerebellar hemisphere removed. Many of the critically important deeper lying nuclei and tracts are projected onto the surface of the lateral brainstem. Note the oculomotor nucleus, the corticospinal tract (CS), trochlear nerve nucleus (IV), superior colliculus (SC), pineal gland (PG), inferior colliculus (IC), trigeminal nerve (V), internal genu (IG), facial/vestibulocochlear complex (VII/VIII), abducens nucleus (A), facial nucleus (F), superior cerebellar peduncle (SP), nucleus ambiguus (NA), hypoglossal nucleus (XII), and dorsal vagal nucleus (X). The solitary nucleus is approximated by the dotted line shown for the dorsal vagal nucleus. The horizontal purple line represents the junction between the lower medulla oblongata and spinal cord.

Histological images were used to verify the various structures shown in the *Atlas'* images. The combination of axial, coronal, and sagittal sections were used to identify critically important deeper nuclei and tracts. These structures were then marked on the surface of the lateral brainstem with pins and skin markers (Figure 4).

Based on these surface markings, regions with a smaller concentration of eloquent nervous structures were defined as regions that might result in less patient morbidity if entered for approaches to deeper lying structures of the brainstem. No specimen was known to have diseases or past surgical intervention of the brain or brainstem. No specimen had a cause of death related to the brainstem or brain.

## Results

Two areas of the lateral brainstem were found to have the least concentration of eloquent nuclei/tracts. These were defined as superior acute and inferior obtuse corridors, i.e. surgical entrance sites into the brainstem for the pontomesencephalic and pontomedullary junctions, respectively.

The superior acute corridor (V-shaped) has an anterior limb that travels superior to the middle cerebellar peduncle, parallel to the exit of the trigeminal nerve and just inferior to the pontomesencephalic junction. The two limbs of the superior acute corridor were approximately 1 cm in length each. The posterior limb of this corridor travels superiorly in the groove formed by the anterior crus cerebri and posteriorly located inferior brachium. The two limbs avoid important structures such as the corticospinal tract and the oculomotor and trochlear nuclei. The inferior obtuse corridor has an anterior limb that travels from the pontomedullary junction anteriorly parallel to the VII/VIII complex. The posterior limb of this corridor travels over the trigeminal tubercle avoiding the rootlets of the glossopharyngeal and vagus nerves in the postolivary sulcus. The two limbs of the inferior obtuse corridor were approximately 1.5 cm in length each. The anterior limb avoids the VII/VIII complex and their nuclei and the corticospinal tract. The posterior limb avoids important nuclear columns in the medulla such as the nucleus ambiguus and hypoglossal nuclei and middle cerebellar peduncle. If this limb is less than 3–4 mm in depth, then it also avoids the deeper lying vestibular nuclei. No significant variability was seen between specimens regarding the position and relationship of the internal brainstem structures evaluated and the surface proposed incision sites. The observations and measurements between specimens were very consistent.

### Case illustrations

**Figure 5:**
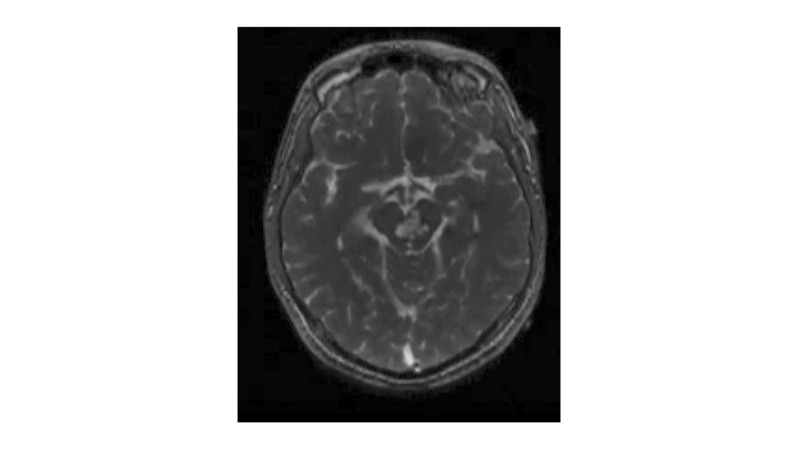
Preoperative MRI of case illustration.

An 18-year-old male presented in 2009 with a six-year history of repeated hemorrhage from a brainstem cavernous malformation (Figure 5).

**Figure 6:**
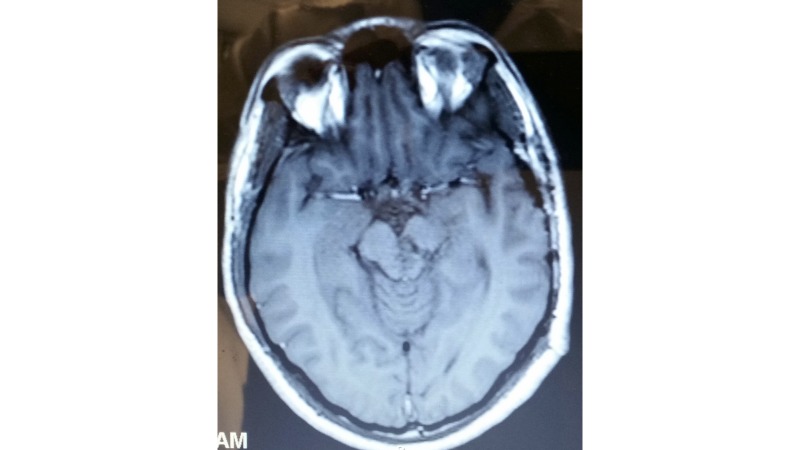
Postoperative MRI of case illustration.

At his most recent presentation, he developed repeated episodes of temporary right hemiparesis and decreased fluency of speech. The lesion was removed via a lateral brain stem approach. The brain stem was opened using the midbrain entry site along the posterior half of the incision shown in Figure 4. The postoperative magnetic resonance imaging (MRI) of the case illustration is shown in Figure 6.

Postoperatively, he continued to have mild speech fluency deficits. His motor examination was 5/5 in all major muscle groups tested. Other than his preoperative speech fluency deficit, no new neurological deficits were identified on physical examination during the postoperative period. This case underwent motor evoked potentials (MEP), somatosensory evoked potentials (SSEP), and brainstem auditory evoked responses (BAER) monitoring. Cranial nerves 3-7 were monitored and a monoprobe was used for all nerve stimulation.

## Discussion

It is well accepted that despite diffuse brainstem gliomas, other types of brainstem tumors might be amenable to surgery; however, due to the anatomical complexity in this area the case of surgery should be individualized for each patient. The fact that neural structures in the region are mainly deviated and not infiltrated by the tumor, makes resection less risky, especially for focal brainstem tumors. In cadavers, we have established surface maps of the more deeply located eloquent nuclei and tracts of the brainstem. Although multiple variables might alter the desired trajectory (e.g. arterial perforators, tumor displacement of surrounding neural structures), these landmarks will give the neurosurgeon additional guidance as to where to make their incision into the brainstem and to do so in areas that are less dense with eloquent neural structures. We accept that there is no perfect area to transect the brainstem. However, if an intramedullary entrance is necessary, less damage might result using these guidelines.

There are some authors who have discussed lateral approaches to the brainstem. For example, tumors of the lateral aspect of the brain stem in children were approached by Vougioukas, et al. using a far lateral supracerebellar infratentorial approach [6]. The authors supported the utility of this approach as no new neurological deficits were found postoperatively in 85.7% of patients although no brainstem landmarks were mentioned in relation to the safe resection of the tumors. Furthermore, the far lateral approach, without condylar resection, has been used to resect lateral brainstem tumors [7-9]. However, such papers, again, did not provide anatomical details for the intramedullary tumor resection.

The far lateral approach provides sufficient access to pathology anterolateral to the brainstem. Hence, safe entry points into the lateral brainstem as described in the present study are particularly desirable. Such entry points must involve areas containing less dense areas of important neural structures. However, as illustrated above, few studies have examined the best corridors into the brainstem, in general. For posterior approaches to the brainstem through the floor of the fourth ventricle, Kyoshima, et al. have described injuries to various brainstem nuclei and tracts as tolerable and “acceptable.” They defined the disability incurred after injury to the following structures as probably manageable: vestibular nuclei, cerebellar peduncles, lateral lemniscus, cochlear nuclei, and the sensory trigeminal nuclei. Structures that were thought to be important not to be injured during such approaches included the lower cranial nerve nuclei, corticospinal tract, corticonuclear tract, parapontine reticular formation, facial, abducens and oculomotor nuclei, and the medial longitudinal fasciculus [10-11]. Additionally, a safe approach to the ventral midbrain was previously described by Cantore, et al. [12]. This relatively safe approach is based on the fact that the fibers of the corticospinal tract occupy only the intermediate three-fifths of the peduncle [13]. The entry point described by these authors was the posterior cerebral artery as the superior border, the superior cerebellar artery as the inferior border, and the emergence of cranial nerve III and the basilar artery as the medial border while the lateral aspect was bordered by the pyramidal tract.

In their study of anterolateral safe entry zones to the brain stem, Recalde, et al. mentioned an approach via the lateral mesencephalic sulcus in the anterolateral mesencephalon [14]. This zone is bounded ventrally by the substantia nigra, dorsally by the medial lemniscus and medially by the fibers of cranial nerve III. They described a safe myelotomy up to nearly 1 cm in length and dissection preferably up to 4.9 mm deep. This entry also poses little risk to important regional perforators [14-15]. These authors found that the transverse plane where the trigeminal nerve exits the pons has an area of low density of motor fibers about 1 cm wide and 1 cm from the midline [12-13]. Lateral widening to the root of cranial nerve V can be considered safe up to 5 mm. Bricolo and Turazzi suggested that, for cavernous angiomas situated in the dorsal midbrain roof that dip into the fourth ventricle, an approach can be taken through the cerebellomedullary fissure by elevating and spreading the cerebellar tonsils using a trans-tela choroidea approach [13]. This minimizes the possibility of injury to the colliculi [12].

Oiwa, et al. used an incision rostral to the VII-VIII complex to remove an intramedullary hematoma [16] and Hauck, et al. utilized this area for removal of a pontine cavernoma [17]. However, based on our study, such an incision would put the internal genu at substantial risk of being injured. The boundaries of this peritrigeminal triangular area are as follows: the pyramidal tract medially, the pontomedullary sulcus (from the lateral aspect of the pyramid to the flocculus) forming the base, and a line from the most medial point of the pontomesencephalic sulcus to the trigeminal nerve forming the lateral limit. For optimal safety, the surgeon should stay within a 3.8 mm horizontal boundary and a 9.5 mm vertical boundary [15].

Medullary safe zones are uncommonly reported because of the density of vital structures located within. However, Recalde, et al. described the retro-olivary sulcus as the zone of safest entry into the anterolateral medulla [14]. The olivary body provides a “safe” window measuring 13.5 mm craniocaudally, 7 mm in the transverse plane, and 2.5 mm in the anterodorsal axis.

Kyoshima, et al. treated three patients with localized intramedullary tumors [18]. The first patient suffered from a vascular malformation extending from the caudal pons to the midbrain and was treated via the suprafacial colliculus approach [19]. The infrafacial approach was used in a second patient who suffered from metastatic renal cell carcinoma in the lower pons. With an intraoperative diagnosis of squamous cell carcinoma by biopsy, the third patient was also treated via the suprafacial approach. This lesion extended from the left pons to the midbrain. The patients that underwent the suprafacial approach experienced medial gaze disturbance and abducens nerve paresis. After the infrafacial approach, the patient experienced temporary facial palsy and swallowing disturbance, lateral gaze disturbance to the right, and hemisensory disturbance for touch or position together with ataxia. These adverse outcomes were believed to due to retraction, en bloc tumor removal or, in the case of the swallowing impairment, extension of the incision over the caudal edge of the striae medullares or damage to the corticonuclear tracts. The suprafacial triangle is bordered medially by the medial longitudinal fascicle, caudally by the internal genu of the facial nerve, and laterally by the superior and inferior cerebellar peduncles. Deep to this triangle are the medial lemniscus and the corticospinal tract. A longitudinal incision, up to 1 cm in length, caudal from the edge of the cerebellar peduncle and about 5 mm lateral to the median sulcus provides entry into this safe triangle [16, 18, 20-22]. Bogucki, et al. [20] found the facial colliculus to be poorly visible in 37.5% of their specimens. They suggested that, in such instances, the distance from the obex to the rostral border of the facial colliculus might be of use in cases where the floor of the fourth ventricle is not distorted by pathology.

The smaller infrafacial triangle for posterior medullary incisions is bordered medially by the medial longitudinal fascicle, caudally by the striae medullares and laterally by the facial nerve. Again, deep to this triangle are the medial lemniscus and the corticospinal tract. A longitudinal incision, rostral from the caudal margin of the striae medullares and about 5 mm lateral to the median sulcus provides the most reasonable entry point into this triangle. Again, this incision should be less than 1 cm [16, 18, 22]. In cases where the striae medullares is not sufficiently visible, the rostral margin of the hypoglossal triangle can serve as an alternative caudal border [20-21].

## Conclusions

“Safe access” points in the brainstem might assist the neurosurgeon in minimizing postoperative morbidity. To our knowledge, a superficial map of the lateral brainstem for identifying deeper lying and clinically significant nuclei and tracts as shown in our study has not previously been available. Such data might decrease patient morbidity following biopsy or tumor removal or aspiration of brainstem hemorrhage; however, due to the possible anatomical distortion by the tumor, these data should be verified by intraoperative electrophysiological neuromonitoring. Also, regional blood vessels must be taken into account. For example, the upper incisions described in our study would be near the posterior choroidal and quadrigeminal arteries. For the lower incisions proposed in our study, the anterior inferior cerebellar artery and its branches would be nearby. These branches and others must be avoided. Additionally, the information from our study can be coupled with the previous literature on approaches into the fourth ventricular floor for more complex, multidimensional lesions.
